# Decoding Glycosylation in Neurodegenerative Diseases: Mechanistic Insights and Therapeutic Opportunities

**DOI:** 10.1096/fj.202502809R

**Published:** 2025-10-19

**Authors:** Haihan Yu, Xing Chen, Yang Yang, Mengke Gu, Kaidi Ren, Ziqing Wei

**Affiliations:** ^1^ Department of Neurology The First Affiliated Hospital of Zhengzhou University Zhengzhou P. R. China; ^2^ Clinical Systems Biology Laboratories The First Affiliated Hospital of Zhengzhou University Zhengzhou P. R. China; ^3^ Department of Pharmacy The First Affiliated Hospital of Zhengzhou University Zhengzhou P. R. China

**Keywords:** glycosylation, neurodegenerative diseases, neuroinflammation, post‐translational modification, therapeutic strategies

## Abstract

Glycosylation is a highly dynamic and complex post‐translational modification that plays a pivotal role in regulating protein folding, trafficking, stability, and function. Accumulating evidence indicates that aberrant glycosylation is intimately involved in the pathogenesis of multiple neurodegenerative diseases, including Alzheimer's disease (AD), Parkinson's disease (PD), Huntington's disease (HD), and amyotrophic lateral sclerosis (ALS). This review provides a comprehensive overview of the molecular mechanisms by which the two predominant forms of glycosylation, N‐glycosylation and O‐GlcNAcylation, contribute to protein misfolding, synaptic dysfunction, neuroinflammation, and impaired stress responses in the diseased nervous system. We further explore the diagnostic potential of glycosylation biomarkers and emerging therapeutic strategies targeting glycosylation pathways. Special emphasis has been placed on recent advances in glycomic technologies, artificial intelligence‐driven analytics, and nanocarrier‐based drug delivery platforms. By integrating mechanistic insights with translational applications, this review highlights glycosylation as both a pathological driver and a promising therapeutic target in neurodegenerative disorders.

## Graphical abstract

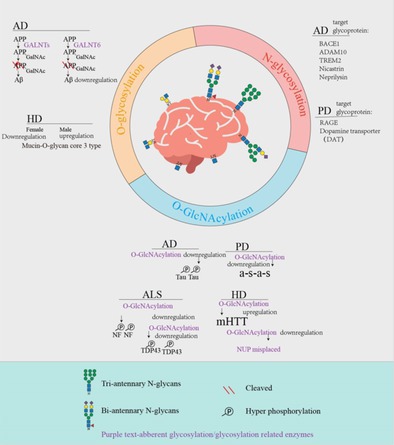

## 1 Introduction

Glycosylation is one of the most common post‐translational modifications of proteins, occurring universally in eukaryotes [1]. This process is essential for proper protein folding, stability, and trafficking [2, 3, 4]. Glycosylation affects a variety of biological functions and physiological processes, including cell adhesion, molecular transport, receptor activation, and signal transduction [4, 5].

Importantly, glycosylation is metabolically connected to glycolysis, the central pathway of glucose catabolism [6]. Through the hexosamine biosynthetic pathway (HBP), a small fraction of glycolytic intermediates (such as fructose‐6‐phosphate) are diverted to generate UDP‐N‐acetylglucosamine (UDP‐GlcNAc) and other activated glycan donors [7]. These nucleotide glycans serve as essential substrates for glycosyltransferases, thereby linking cellular energy and nutrient status to the regulation of glycosylation [6]. This metabolic crosstalk ensures that protein glycosylation is dynamically modulated in response to environmental and physiological conditions.

Protein glycosylation is mediated by complex biosynthetic pathways involving the coordinated interplay of numerous enzymes and organelles [4]. Hundreds of glycosyltransferases and glycosidases, together with transport proteins and scaffolding factors, contribute to the diversity of glycan structures [4, 8]. The broad range of monosaccharide donors and glycosidic linkages results in highly heterogeneous glycoproteins with context‐specific biological functions [9]. In eukaryotic cells, glycoproteins typically undergo initial folding and N‐linked glycosylation in the endoplasmic reticulum (ER), followed by further trimming, extension, and terminal modification in the Golgi apparatus [10]. After processing, glycoproteins are sorted via vesicular transport: some are recycled back to the ER through coat protein I (COPI) vesicles, whereas others are delivered to the plasma membrane, where they participate in cell–cell adhesion, ligand–receptor interactions, pathogen defense, or toxin recognition [11].

Aberrant glycosylation plays a crucial role in the onset and progression of various diseases [6, 12], including cancer, diabetes, immune disorders, infectious diseases, and neurological conditions [13, 14, 15]. In neurodegenerative diseases, abnormal glycosylation contributes to disease progression through multiple mechanisms [16], including promoting the aggregation of pathological proteins (e.g., amyloid‐β, tau, α‐synuclein, and mutant huntingtin), impairing synaptic function, exacerbating neuroinflammation, and disrupting cellular stress responses [6, 13]. These insights provide new perspectives on the pathogenesis of neurodegenerative diseases and lay a theoretical foundation for the development of targeted therapies. Elucidating the mechanisms and roles of glycosylation in disease not only advances our understanding of pathological processes but also offers novel strategies and targets for diagnosis and treatment.

## 2 Types of Glycosylation

**FIGURE 1:**
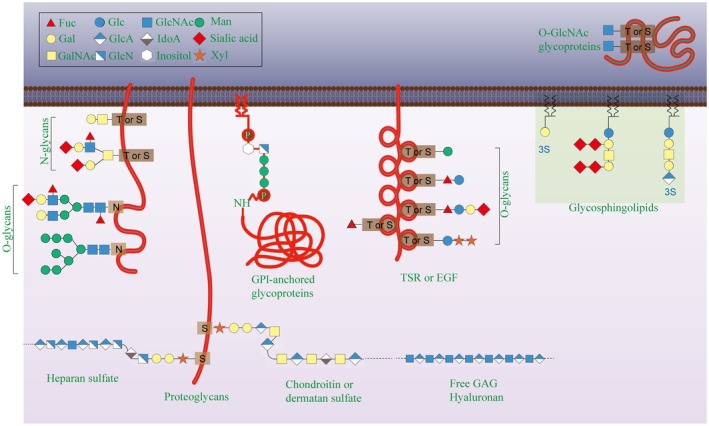
Major forms of protein glycosylation. This schematic illustrates the primary types of covalent glycan attached to proteins. Key structural features are as follows: N‐glycosylation involves the attachment of an oligosaccharide to the amide nitrogen of asparagine (Asn); O‐glycosylation involves the attachment of glycans (e.g., mucin‐type O‐GalNAc) to the hydroxyl oxygen of serine (Ser) or threonine (Thr); O‐GlcNAcylation is a specific monosaccharide modification of Ser/Thr residues; glycosylphosphatidylinositol (GPI) anchors use a glycan bridge to attach proteins to membrane lipids; C‐mannosylation involves direct C‐C attachment of mannose to tryptophan (Trp); and glycosaminoglycans (GAGs) form long linear polysaccharide chains attached to core proteins. The figure was drawn via Adobe Illustrator (Adobe Inc., San Jose, CA, USA).

Protein glycosylation can be classified on the basis of the covalent attachment of carbohydrate chains (glycans) to specific amino acid residues in the protein backbone [17]. The major types of glycosylation include N‐linked glycans, which are attached to the amide nitrogen of asparagine residues [18]; O‐linked glycans, which are attached to the hydroxyl groups of serine or threonine residues [19]; glycosaminoglycans, which are long linear polysaccharides covalently linked to core proteins [20]; glycosylphosphatidylinositol (GPI) anchors, in which glycans connect proteins to lipid moieties [21]; phosphorylated glycans [22]; and C‐mannosylation, where mannose residues are covalently linked to the indole C2 carbon of tryptophan residues [23] (Figure 1).

**FIGURE 2:**
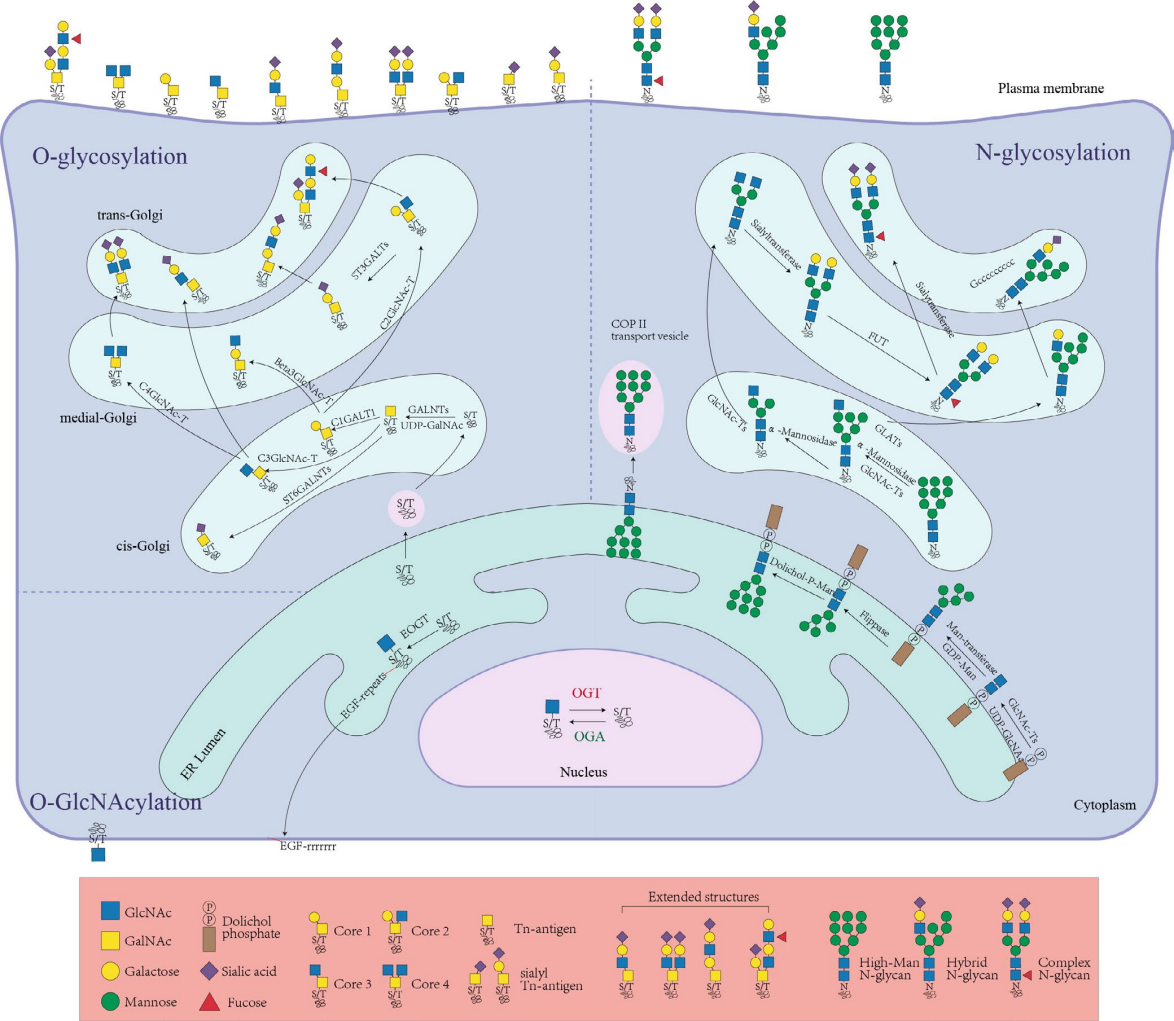
Graphical summary of the comprehensive overview of glycosylation. N‐glycosylation initiates at the outer leaflet of the endoplasmic reticulum (ER), where glycosyltransferases stepwise assemble UDP‐GlcNAc and GDP‐Man into a lipid‐linked oligosaccharide precursor (Glc_3_Man_9_GlcNAc_2_‐PP‐dolichol). This precursor is translocated into the ER lumen, followed by en bloc transfer to asparagine (Asn) residues of nascent polypeptides by oligosaccharyltransferase (OST). In the ER and Golgi apparatus, α‐glucosidases trim glucose residues, whereas mannosidases and glycosyltransferases further modify the glycans to form complex or hybrid N‐glycans. O‐glycosylation predominantly commences in the Golgi apparatus, where polypeptide N‐acetylgalactosaminyltransferases (ppGalNAc‐Ts) conjugate UDP‐GalNAc to serine/threonine (Ser/Thr) residues, forming the Tn antigen (GalNAc‐O‐Ser/Thr). Enzymes such as β3Gal‐T and C1GALT1 sequentially add galactose (Gal), GlcNAc, and other moieties to construct core structures (e.g., Core 1 and Core 2), which are further extended into complex glycans decorated with sialic acid or fucose. O‐GlcNAcylation occurs in the cytoplasm, nucleus, and mitochondria, with UDP‐GlcNAc serving as the glycan donor. O‐GlcNAc transferase (OGT) catalyzes the addition of a single GlcNAc to Ser/Thr residues, whereas O‐GlcNAcase (OGA) mediates the dynamic removal of this modification. The figure was drawn via Adobe Illustrator (Adobe Inc., San Jose, CA, USA).

N‐glycosylation is initiated in the endoplasmic reticulum, where lipid‐linked oligosaccharide precursors are assembled and subsequently transferred to asparagine residues of nascent polypeptides [24] (Figure 2). This process is essential for protein folding, quality control, and transport to the Golgi apparatus for further maturation [25, 26, 27]. O‐glycosylation mainly occurs in the Golgi, beginning with the attachment of N‐acetylgalactosamine to serine or threonine residues, followed by sequential elongation and capping to generate structurally diverse glycans that regulate protein secretion and receptor activity [28](Figure 2). In contrast, O‐GlcNAcylation is a dynamic and reversible modification in the cytoplasm and nucleus, catalyzed by O‐GlcNAc transferase (OGT) and O‐GlcNAcase (OGA) [29] (Figure 2). This unique feature enables O‐GlcNAc to function as a nutrient and stress sensor, rapidly responding to metabolic fluctuations and modulating neuronal processes such as transcription, synaptic plasticity, and stress adaptation [29]. Together, these glycosylation pathways form a regulatory “glycosylation code” that modulates protein function and contributes to disease pathogenesis, including neurodegeneration [30]. Owing to the structural and conformational diversity of glycans, along with various linkage methods, glycosylation is considered one of the most abundant post‐translational modifications in living organisms [4].

### 2.1 N‐Glycosylation

N‐glycosylation is a co‐translational and post‐translational modification in which oligosaccharides are covalently attached to the amide nitrogen of asparagine residues within a consensus sequence (Asn‐X‐Ser/Thr, where X ≠ Pro) [31]. The designation “N” originates from this linkage to the asparagine side‐chain nitrogen [32]. This highly conserved process occurs in virtually all eukaryotes and is essential for proper protein folding, quality control, trafficking, and stability, as well as for mediating cell–cell and cell–matrix interactions [27, 33].

N‐glycosylation begins in the ER with nascent polypeptide chains, where initial processing takes place before the glycoprotein is transferred to the Golgi apparatus for further maturation into complex N‐glycans [34] (Figure 2). The formation of N‐glycans requires glycan donors, including UDP‐GlcNAc, GDP‐mannose (GDP‐Man), and UDP‐glucose (UDP‐Glc), which provide the essential monosaccharide units for precursor assembly and subsequent maturation [35]. Their precursors are synthesized in the ER lumen by a family of asparagine‐linked glycosyltransferases (ALGs), which catalyze the transfer of lipid‐linked oligosaccharides to dolichol phosphate (Dol‐P) embedded in the membrane [27]. This enzymatic series ultimately generates the Glc_3_Man_9_GlcNAc_2_‐PP‐Dol structure, which serves as the donor for N‐glycan assembly [31].

The oligosaccharyltransferase (OST) complex, located on the ER membrane, subsequently catalyzes the covalent attachment of Glc_3_Man_9_GlcNAc_2_ to the amide nitrogen of an asparagine (Asn) residue in the nascent polypeptide chain via a β1 linkage [36]. OST recognizes the Asn‐X‐Ser/Thr motif within the growing polypeptide and transfers the core glycan to the Asn residue. To ensure optimal recognition of the acceptor sequence, this transfer typically occurs before disulfide bond formation in the protein [37].

Following this, the glycoprotein undergoes further trimming in the ER by α‐glucosidases, which remove glucose residues and one mannose residue to generate a GlcNAc_2_Man₈ structure. This intermediate is then transported to the Golgi apparatus, where it undergoes extensive modification and elongation by a series of glycosyltransferases and glycosidases, resulting in three major types of N‐glycans: high‐mannose, complex, and hybrid structures [38]. The terminal residues of these complex N‐glycans may include N‐acetylglucosamine, N‐acetylgalactosamine, sialic acid, and fucose [24]. These terminal structures determine glycan classification and play essential roles in modulating protein conformation, antigenicity, biological activity, and molecular recognition [18].

### 2.2 O‐Glycosylation

In eukaryotic cells, two common forms of O‐glycosylation are O‐GalNAc (mucin‐type O‐glycosylation) and O‐GlcNAc (O‐linked β‐D‐N‐acetylglucosamine), both of which involve the attachment of glycan moieties to the hydroxyl groups of serine or threonine residues [39].

O‐GalNAc typically appears in clusters [28]. Owing to the abundance of serine/threonine motifs in mucins that serve as recognition sites for O‐GalNAc addition, this modification is also referred to as mucin‐type O‐glycosylation [40]. Mucin‐type O‐glycosylation occurs in the Golgi apparatus and involves more than 20 members of the polypeptide N‐acetylgalactosaminyltransferase (GALNT) family [40]. These enzymes transfer N‐acetylgalactosamine (GalNAc) from UDP‐GalNAc to Ser or Thr residues, forming the Tn antigen (GalNAc‐O‐Ser/Thr) (Figure 2). C1GalT1 subsequently extends the Tn antigen by adding galactose to produce the core 1 structure (T antigen). Under the catalysis of various core‐specific glycosyltransferases, the Tn and T antigens are elaborated into multiple O‐glycan structures [41]. These core glycans may be further extended with galactose, fucose, or sialic acid through α‐ or β‐glycosidic bonds to form linear or branched structures [40]. Finally, terminal capping of O‐GalNAc‐modified proteins is achieved mainly via sialylation by ST3GAL and ST6GAL sialyltransferases, which occurs progressively from the cis‐ to the trans‐Golgi network [28]. Certain O‐GalNAc secretory proteins, such as MUC5AC, are ultimately packaged into vesicles for final secretion [42].

### 2.3 O‐GlcNAclation

O‐GlcNAc is a dynamic and reversible posttranslational modification involving the addition of a single N‐acetylglucosamine (GlcNAc) residue via an O‐linkage. This modification occurs extensively in the cytoplasm, nucleus, and mitochondria of eukaryotic cells [39]. Owing to its simplicity and reversibility, O‐GlcNAc can rapidly respond to extracellular stimuli and flexibly modulate the intracellular signaling pathway [43]. It is widely present throughout brain development and is distinct from classical phosphorylation. O‐GlcNAcylation is exclusively regulated by two enzymes: OGT and OGA [44, 45]. OGT facilitates the transfer of GlcNAc from UDP‐GlcNAc to serine or threonine residues on substrate proteins, whereas OGA removes GlcNAc residues from these proteins [46]. UDP‐GlcNAc, the donor substrate for O‐GlcNAc, is synthesized via the HBP from glucose [46, 47]. As a nutrient sensor and a central modulator of transcription, signaling, and metabolism, O‐GlcNAc contributes to stress adaptation, metabolic regulation, enzyme activity, and subcellular localization [48].

## 3 Glycosylation and Neuronal Cell Biology

Glycosylation plays a multifaceted regulatory role in neuronal cell biology, significantly influencing various physiological and pathological processes [6, 49, 50]. Aberrant glycosylation contributes to disease progression through multiple mechanisms, including the promotion of pathological protein aggregation [25, 51, 52], disruption of synaptic function [53, 54], exacerbation of neuroinflammation [15], and impairment of cellular stress responses [55].

### 3.1 Impact on Protein Misfolding and Aggregation

The glycosylation of specific proteins is essential for maintaining their normal biological function. In neurodegenerative diseases, the glycosylation of these proteins is frequently altered, affecting their stability, solubility, and functional activity [13, 16, 54]. N‐glycosylation plays a key role in protein folding, stability, and trafficking [26]. For example, glycosylation of amyloid precursor protein (APP), which is implicated in Alzheimer's disease (AD), influences its processing and the subsequent formation of amyloid‐beta (Aβ) plaques [56]. O‐glycosylation is also critical for modulating protein–protein interactions, including those essential for synaptic function [57].

Glycosylation can influence the folding, trafficking, and aggregation of these proteins [58, 59]. In some neurodegenerative conditions, abnormal glycosylation patterns may increase the likelihood of protein misfolding or aggregation, whereas in others, glycosylation may exert a protective effect [60].

In AD, changes in APP glycosylation affect its cleavage by β‐ and γ‐secretases, leading to the generation of Aβ peptides that aggregate into toxic plaques [61]. These plaques are neurotoxic and contribute to neuroinflammation and synaptic dysfunction [62]. Moreover, glycosylation of tau, another key protein in AD pathogenesis, influences its phosphorylation and the formation of neurofibrillary tangles—another hallmark of the disease [63]. In Parkinson's disease (PD), the glycosylation of α‐synuclein, the principal component of Lewy bodies, modulates its aggregation and toxicity [58]. Specific glycosylation patterns of α‐synuclein can increase its tendency to form insoluble aggregates, leading to the degeneration of dopaminergic neurons in the substantia nigra [58].

### 3.2 Glycosylation and Synaptic Function

Glycosylation plays crucial roles in synaptic plasticity, neurotransmitter release, and receptor function [64]. Glycoproteins are abundantly present in synaptic vesicles, with over 2500 glycoproteins identified in synapses and synaptic vesicles in mice [65]. Altered glycosylation of synaptic proteins can impair synaptic communication, resulting in cognitive deficits and neurodegeneration [65, 66]. Highly fucosylated glycans are found primarily on synaptic vesicle proteins, cell adhesion molecules involved in synaptic function, and membrane proteins related to protein and neurotransmitter transport [65].

Synaptic vesicle protein 2 (SV2) contains multiple N‐glycan chains, and its function is highly dependent on N‐glycosylation. N‐glycan modifications on multiple asparagine residues are essential for SV2 trafficking and synaptic vesicle formation [67]. Abnormal O‐glycosylation of the tau protein promotes its hyperphosphorylation and aggregation, thereby disrupting synaptic function and contributing to neurodegeneration in AD and other tauopathies, such as frontotemporal dementia [68]. The glycosylation of ionotropic glutamate receptors, such as AMPA and NMDA receptors, regulates their trafficking to the synaptic membrane and their roles in synaptic plasticity [69, 70]. Dysregulated glycosylation of these receptors leads to synaptic dysfunction—a hallmark of various neurodegenerative diseases, including AD and PD.

### 3.3 Glycosylation, the Immune Response, and Neuroinflammation

Neuroinflammation is a common feature of neurodegenerative diseases and is believed to contribute to disease progression [71, 72]. Glycosylation plays a key role in modulating immune responses, including the activation of microglia (resident immune cells of the brain) and astrocytes [29, 73]. Altered glycosylation of cell surface receptors affects the activation states of these glial cells, leading to excessive inflammation and exacerbation of neuronal damage [29, 73]. Glycosylation changes in surface proteins such as Toll‐like receptors (TLRs) can increase microglial activation, promoting the release of pro‐inflammatory cytokines and reactive oxygen species (ROS) [74, 75]. Chronic neuroinflammation, in turn, worsens neurodegeneration by increasing oxidative stress and impairing synaptic function [76]. Glycosylation also regulates the adhesion and migration of immune cells to sites of injury [77, 78]. Changes in the glycosylation of adhesion molecules such as integrins and selectins influence neuroinflammatory processes in neurodegenerative diseases [78].

### 3.4 Glycosylation and Cellular Stress Responses

Cellular stress responses, including the unfolded protein response (UPR) and oxidative stress pathways, are essential for maintaining neuronal homeostasis [79]. Dysregulated glycosylation can impair these pathways, leading to cellular damage and death. Altered glycosylation within the endoplasmic reticulum (ER) can disrupt protein folding and trafficking, resulting in ER stress [80]. In neurodegenerative diseases such as AD and PD, misfolded proteins (e.g., Aβ, tau, and α‐synuclein) accumulate in the ER, triggering UPR activation. Chronic ER stress can lead to neuronal apoptosis and further neurodegeneration [81].

Glycosylation also influences the expression of antioxidant proteins and the cellular response to oxidative stress [82]. In diseases such as Huntington's disease (HD), oxidative stress is a major contributor to neuronal injury, and altered glycosylation of proteins involved in antioxidant defense may impair the ability of the brain to cope with ROS [83].

In summary, aberrant glycosylation can lead to neuronal dysfunction, toxic protein aggregation, and inflammation—hallmarks of neurodegenerative diseases. Elucidating the role of glycosylation in these disorders provides new insights into pathogenesis and offers potential therapeutic targets aimed at modulating glycosylation patterns to prevent or slow disease progression.

## 4 The Role of Glycosylation in Neurodegenerative Diseases

In the brain, glycosylation is involved in various aspects of neuronal development, signal transduction, and synaptic plasticity [84, 85]. However, alterations in glycosylation can significantly contribute to the pathogenesis of neurodegenerative diseases by modifying protein function, disrupting cellular signaling, and exacerbating inflammatory responses [84]. Neurodegenerative diseases such as AD, PD, Huntington's disease (HD), and amyotrophic lateral sclerosis (ALS) are characterized by progressive neuronal degeneration, often accompanied by abnormal protein aggregation [86], inflammation [72], and cellular dysfunction [87]. Increasing evidence suggests that glycosylation abnormalities play a pivotal role in the pathophysiology of these disorders [84] (Table 1).

**TABLE 1:** Major types of protein glycosylation and their relevance in neurodegenerative diseases.

Glycosylation type	Structural feature	Physiological significance	Pathological role in neurodegenerative diseases
N‐glycosylation	Attachment of glycans to the amide nitrogen of asparagine (Asn‐X‐Ser/Thr motif)	Ensures proper protein folding, stability, and trafficking; regulates cell–cell communication	Aberrant N‐glycosylation promotes misfolding/aggregation of amyloid‐β, tau, and α‐synuclein; implicated in AD, PD, ALS [25, 26, 88]
O‐glycosylation	Attachment of glycans to the hydroxyl group of serine/threonine	Modulates protein solubility, secretion, and receptor activity	Dysregulated O‐glycosylation alters receptor signaling and extracellular matrix interactions, potentially aggravating neurodegeneration [28, 51]
O‐GlcNAcylation	Addition of single N‐acetylglucosamine (GlcNAc) to serine/threonine residues in nuclear and cytoplasmic proteins	Dynamic and reversible modification regulating transcription, signal transduction, and stress responses	Competes with phosphorylation on tau, α‐synuclein, and TDP‐43; reduced O‐GlcNAcylation promotes abnormal phosphorylation and aggregation in AD, PD, and ALS [89, 90, 91, 92]
Glycosaminoglycans (GAGs)	Linear polysaccharides (e.g., heparan sulfate, chondroitin sulfate) covalently attached to core proteins	Structural components of extracellular matrix; regulate growth factor signaling	Heparan sulfate proteoglycans interact with amyloid‐β and tau, accelerating aggregation in AD [93]
GPI anchor	Glycan linkage attaching proteins to cell membrane via phosphatidylinositol	Localizes proteins to lipid rafts; involved in signal transduction	Altered GPI‐anchored protein distribution affects prion protein processing in prion diseases [21]
C‐mannosylation	Covalent attachment of mannose to the indole C2 of tryptophan residues	Stabilizes protein folding and secretion	Dysregulation may impair secretion of neuroprotective factors; potential link to AD and other disorders [23]
Phosphoglycosylation	Addition of sugar‐phosphate groups to specific amino acids	Involved in lysosomal enzyme targeting	Defects contribute to impaired lysosomal function, relevant to protein aggregation in AD and PD [94]

### 4.1 Role of Glycosylation in Alzheimer's Disease (AD)

AD is a neurodegenerative disorder characterized by progressive cognitive decline [95]. Its hallmark pathological features include the accumulation of β‐amyloid (Aβ) plaques and the formation of neurofibrillary tangles composed of hyperphosphorylated tau protein [96]. A growing body of evidence indicates a close link between AD and glucose metabolism, leading some researchers to refer to AD as “type 3 diabetes” [97]. As a key post‐translational modification, glycosylation plays a critical role in the pathological processes of AD [98].

Aβ is a peptide fragment generated from amyloid precursor protein (APP) through sequential cleavage by β‐secretase and γ‐secretase [99, 100]. N‐glycosylation of APP influences its processing; studies have shown that increased N‐glycosylation increases the efficiency of β‐secretase (BACE1) cleavage, thereby promoting Aβ production [101]. Furthermore, N‐glycosylation of Aβ may increase its hydrophobicity and aggregation propensity, accelerating amyloid plaque formation [102]. The accumulation of amyloid plaques triggers neurotoxicity, synaptic dysfunction, and neuroinflammation, ultimately leading to neuronal death [103]. Other Aβ‐related molecules, including Nicastrin (a γ‐secretase subunit) [104], ADAM10 (a disintegrin and metalloproteinase) [105], neprilysin (an Aβ‐degrading enzyme) [106], and TREM2 (triggering receptor expressed on myeloid cells 2) [107, 108], have also been reported to undergo N‐glycosylation. However, the underlying mechanisms of aberrant glycosylation of these proteins and their potential contributions to disease pathology remain poorly understood [109].

Similarly, O‐GlcNAc can modulate Aβ production by influencing APP trafficking and cleavage [110]. O‐GlcNAc modification at Thr576 of APP regulates its transport to the plasma membrane and suppresses the generation of the Aβ40 and Aβ42 peptides [111]. The OGA inhibitor Thiamet‐G (TMG) has been shown to reduce Aβ42 production and the amyloid plaque burden in the hippocampus and cortex of a mouse model [112]. Research further indicates that O‐GlcNAcylation suppresses BACE1 activity, thereby reducing Aβ production [113]. In patients with AD, decreased levels of O‐GlcNAc correlate with increased BACE1 activity and elevated Aβ levels, facilitating plaque formation [89]. γ‐Secretase consists of four subunits, and O‐GlcNAc at Ser708 of one subunit inhibits its enzymatic activity [112]. In AD mouse models, the inhibition of OGA reduces Aβ accumulation and neuroinflammation and alleviates memory impairment by increasing the level of O‐GlcNAc at Ser708 [112]. Notably, OGA inhibition in these models increases O‐GlcNAc levels, decreases Aβ40 and Aβ42 levels, reduces plaque formation, and improves cognitive function [114].

**FIGURE 3:**
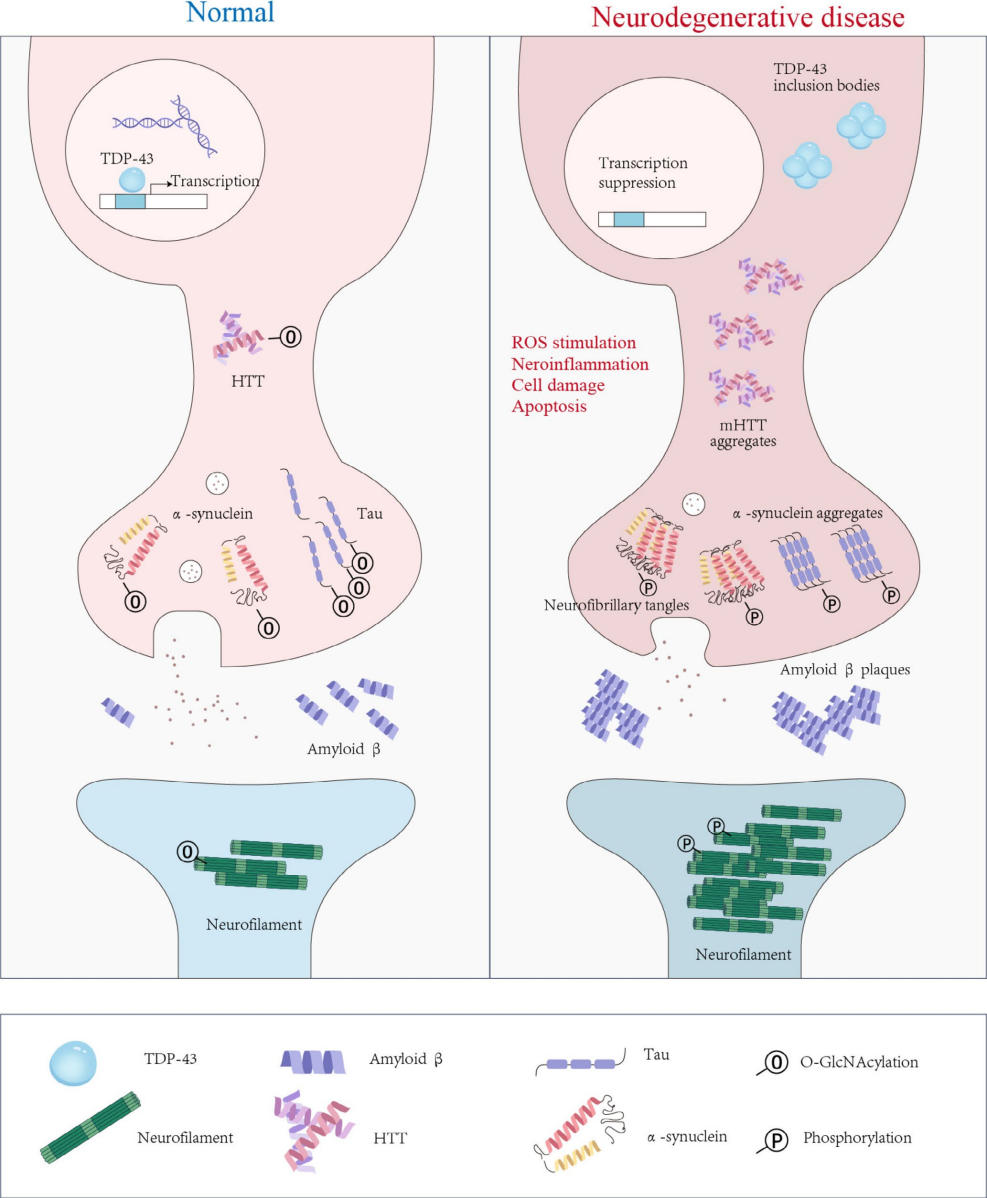
O‐GlcNAcylation and its neuroprotective mechanisms in neurons. O‐GlcNAcylation plays a crucial neuroprotective role by modulating key etiological proteins involved in neurodegenerative diseases. Pathological proteins such as tau, α‐synuclein, huntingtin (HTT), neurofilaments, amyloid‐beta (Aβ), and TDP‐43 can be modified and regulated by O‐GlcNAcylation. In neurodegenerative disorders, dysregulation of O‐GlcNAcylation is frequently observed. This aberrant O‐GlcNAc modification, often accompanied by hyperphosphorylation, promotes the aggregation of pathological proteins, ultimately leading to neuronal toxicity and disease progression. The figure was drawn via Adobe Illustrator (Adobe Inc., San Jose, CA, USA).

Tau, a microtubule‐associated protein responsible for maintaining cytoskeletal stability in neurons, becomes abnormally hyperphosphorylated and aggregates into neurofibrillary tangles (NFTs) in AD [115] [116]. N‐glycosylation promotes tau hyperphosphorylation, whereas removal of this modification reduces tau phosphorylation and alters tangle morphology [117]. High levels of N‐glycosylated tau are found within NFTs, and this modification may contribute to oxidative stress, potentially triggering NFT formation [117]. Early studies also identified O‐GlcNAc on tau, with an inverse correlation between O‐GlcNAc and tau phosphorylation [90]. Under normal conditions, O‐GlcNAc maintains tau solubility and function, promoting microtubule assembly and intracellular transport. Under pathological conditions, reduced O‐GlcNAc leads to tau hyperphosphorylation and NFT formation [117] (Figure 3). In the brains of AD patients, O‐GlcNAc levels are significantly lower than those in healthy individuals and progressively decline with age [118]. In AD mouse models, increased tau phosphorylation coincides with decreased O‐GlcNAc [119, 120]. Furthermore, neuron‐specific knockout of OGT results in increased tau phosphorylation. Treatment with the OGA inhibitor TMG increases O‐GlcNAc at Ser400 of tau, rapidly suppresses tau phosphorylation, and directly inhibits tau aggregation [121]. These findings suggest that increasing O‐GlcNAc levels via OGA inhibition has therapeutic potential for mitigating AD pathology and preventing dementia.

### 4.2 Role of Glycosylation in Parkinson's Disease (PD)

PD is a progressive neurodegenerative disorder primarily characterized by motor dysfunction [122]. The main pathological hallmarks of PD include the abnormal aggregation of α‐synuclein into Lewy bodies and the loss of dopaminergic neurons [123, 124]. N‐glycosylation of α‐synuclein may influence its conformation and aggregation properties [125]. Studies have shown that N‐glycosylation enhances the hydrophobicity of α‐synuclein, promoting its oligomerization and fibrillation [125]. The aberrant aggregation of α‐synuclein into Lewy bodies impairs dopaminergic neuronal function [126]. Furthermore, increased levels of N‐glycosylated α‐synuclein have been observed in the brain tissues of PD patients [127].

Mitochondrial dysfunction is a key pathological feature of PD, contributing to impaired energy metabolism and neuronal death in dopaminergic neurons [128]. Abnormal N‐glycosylation of mitochondrial proteins leads to mitochondrial dysfunction and elevated oxidative stress [129].

Several PD‐related proteins exhibit abnormal glycosylation patterns, which may contribute to disease pathogenesis. The receptor for advanced glycation end products (RAGE) contains two N‐glycosylation sites that modulate the signaling activity by regulating molecular interactions [130]. Additionally, the dopamine transporter (DAT) undergoes N‐glycosylation, which is critical for its cell surface localization and dopamine reuptake function [131].

Under physiological conditions, O‐GlcNAc maintains the solubility and function of α‐synuclein, thereby supporting normal neuronal activity [132]. In PD patients, decreased levels of O‐GlcNAcylation increase the propensity of α‐synuclein to aggregate into Lewy bodies [133]. In PD mouse models, conditional knockout (cKO) of OGA specifically in dopaminergic neurons results in elevated O‐GlcNAc levels, reduced maturation of α‐synuclein aggregates, and significant neuroprotection without detrimental effects on neuronal structure over prolonged periods [91, 134]. Conversely, OGT cKO has harmful effects, indicating that O‐GlcNAc is essential for the health and survival of dopaminergic neurons [91]. Pharmacological upregulation of O‐GlcNAc via Thiamet‐G (ThG) has been shown to attenuate α‐synuclein‐induced neurotoxicity and exert neuroprotective effects [133].

### 4.3 Role of Glycosylation in Huntington's Disease (HD)

HD is a fatal neurodegenerative disorder characterized by motor dysfunction, cognitive decline, and psychiatric symptoms. It is caused primarily by the abnormal expansion of CAG trinucleotide repeats in the huntingtin (HTT) gene [135]. The pathological features of HD include selective neuronal loss in the striatum and cerebral cortex, as well as the formation of mutant huntingtin (mHTT) aggregates [136, 137]. Owing to polyglutamine (polyQ) expansions resulting from CAG repeat elongation, aberrant glycosylation of mHTT directly contributes to disease pathology [138].

Studies have identified multiple potential N‐glycosylation sites on mHTT. For example, glycosylation at Asn422 regulates mHTT stability, whereas glycosylation at Asn538 influences the formation of mHTT aggregates [139, 140]. Abnormal N‐glycosylation induces conformational changes in mHTT, promoting β‐sheet formation and aggregation [26]. Misfolded mHTT accumulates in the endoplasmic reticulum (ER), triggering UPR and ER stress, which ultimately leads to neuronal apoptosis [137, 141]. Moreover, impaired N‐glycosylation may reduce the efficiency of mHTT degradation via the autophagy–lysosomal pathway [142].

Comprehensive glycomic analysis of transgenic mouse models of HD revealed significant sex‐dependent variations in neural tissue glycosylation profiles. Among the most prominently affected structures were core 3‐type mucin O‐glycans (GlcNAcβ1‐3GalNAcα‐Ser/Thr), which demonstrated sexually dimorphic regulation, with elevated levels in male subjects but a reduction in their female counterparts [143]. These findings suggest that sex‐specific modifications in mucin‐type O‐glycosylation may serve as potential diagnostic indicators reflecting sex‐divergent pathological mechanisms in HD progression.

To date, the relationship between O‐GlcNAcylation and HD remains unclear. Notably, O‐GlcNAc modifications have been detected on HTT and HIP1R, implicating O‐GlcNAcylation as a potential regulatory mechanism for HTT function [91]. By competitively inhibiting phosphorylation, O‐GlcNAc reduces the formation of toxic mHTT aggregates. Reduced OGT activity may exacerbate HD pathology. Additionally, O‐GlcNAc modification modulates glutamate receptor function, attenuating excitotoxicity and improving synaptic dysfunction in HD models [144, 145]. mHTT can interfere with the O‐GlcNAc patterns of transcription factors, whereas OGT overexpression partially restores gene expression profiles. Abnormal glucose metabolism in HD may lead to reduced O‐GlcNAc, further aggravating neurodegeneration. Experimental studies have shown that OGT activators such as Thiamet‐G increase global O‐GlcNAc levels, reduce mHTT aggregation, and increase neuronal survival [146]. OGA inhibitors such as MK‐8719 have demonstrated therapeutic efficacy in HD animal models, highlighting the neuroprotective potential of glycosylation [147, 148].

Multiple nucleoporins (NUPs) within the nuclear pore complex (NPC) have been identified as substrates for O‐GlcNAc modification [149]. mHTT can reduce the level of O‐GlcNAcylation [150]. Insufficiently O‐GlcNAcylated NUPs (such as Nup62) are prone to colocalize with mHTT aggregates, forming perinuclear inclusions and leading to structural disorders and nuclear‐cytoplasmic transport disorders in NPCs, exacerbating neuronal dysfunction [150].

Glycosphingolipid (GSL)‐associated glycosylation is also implicated in HD [151]. mHTT may disrupt GSL metabolism, impair lipid raft integrity, and interfere with membrane receptor signaling (e.g., TrkB and IGF‐1R). Aberrant GSLs activate microglia and trigger neuroinflammatory responses.

Thus, glycosylation plays a dual role in HD: physiological modifications (e.g., O‐GlcNAc) may exert protective effects by suppressing mHTT toxicity, whereas pathological modifications (e.g., aberrant N‐glycosylation or disrupted GSL metabolism) promote neurodegeneration. Elucidating the regulatory mechanisms of these modifications may provide novel biomarkers and therapeutic targets for HD.

### 4.4 Role of Glycosylation in Amyotrophic Lateral Sclerosis (ALS)

Amyotrophic lateral sclerosis (ALS) is a fatal neurodegenerative disorder that primarily affects motor neurons, leading to progressive muscle atrophy and paralysis [152, 153]. Approximately 10% of ALS cases are familial (fALS), whereas the remaining 90% are sporadic (sALS) [153]. The major pathogenic proteins implicated in ALS include superoxide dismutase 1 (SOD1) [154], TAR DNA‐binding protein 43 (TDP‐43) [155], and fused in sarcoma (FUS) [156]. Glycosylation participates in ALS pathogenesis by regulating the stability, aggregation propensity, and function of these proteins.

SOD1 is a copper/zinc superoxide dismutase; the mutants, such as SOD1‐G93A, misfold and form toxic aggregates in ALS [154, 157]. While wild‐type SOD1 lacks N‐glycosylation sites, specific mutations introduce novel N‐glycosylation motifs [158]. Glycosylation enhances SOD1 stability and reduces misfolding; however, excessive glycosylation may interfere with enzymatic activity [158]. Glycosylation defects in mutant SOD1 lead to its accumulation in the endoplasmic reticulum (ER), triggering the unfolded protein response (UPR) and promoting motor neuron apoptosis [159, 160]. Molecular chaperones, such as HSP70, facilitate correct folding and reduce toxic aggregate formation [161]. Inhibitors of N‐glycosylation enzymes, such as tunicamycin, have shown partial efficacy in ameliorating pathological phenotypes in ALS models [162, 163].

**FIGURE 4:**
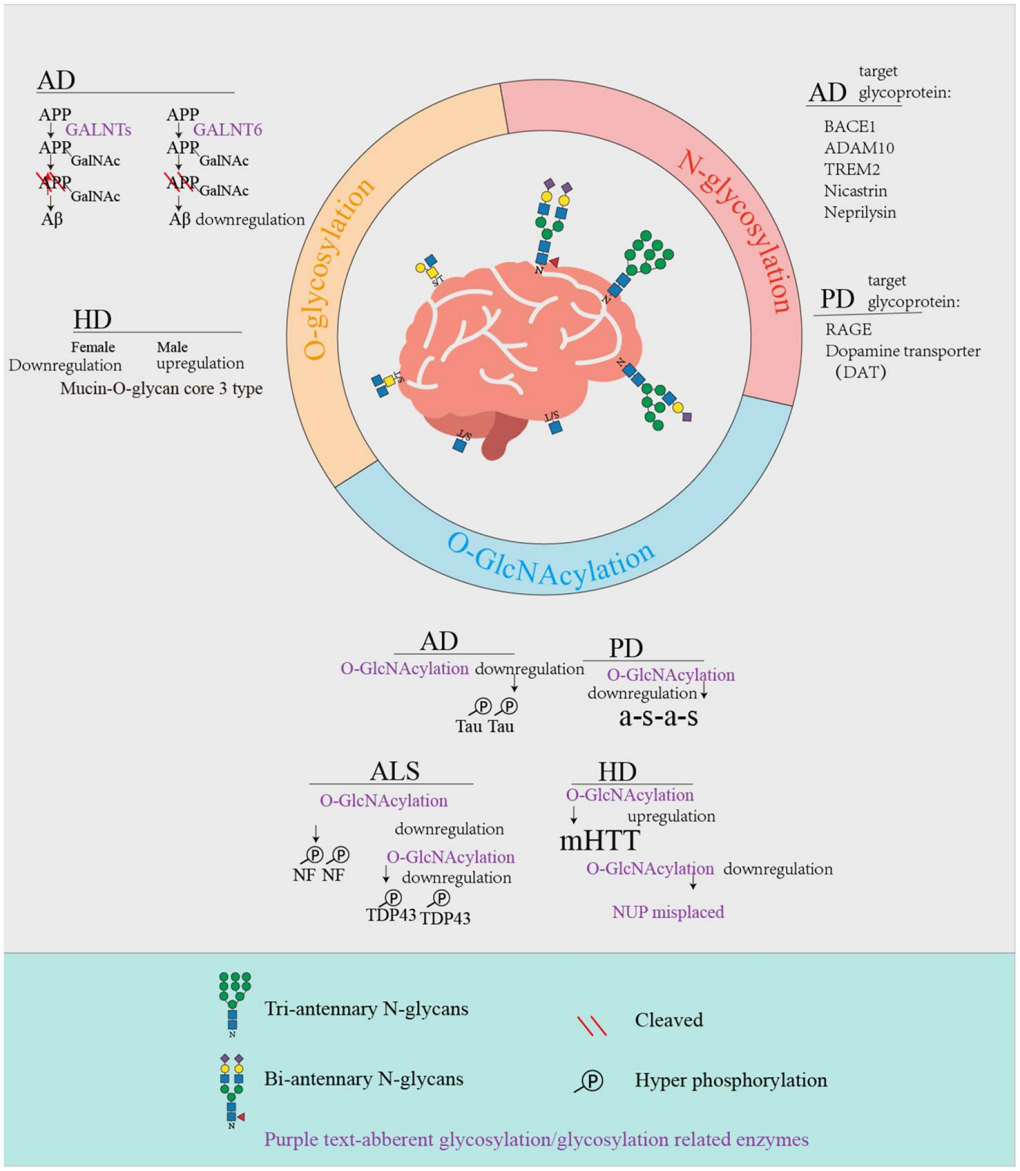
Graphical summary of glycosylation impairments in various neurodegenerative disorders. Among the diverse forms of glycosylation, the most extensively studied include N‐glycosylation, O‐glycosylation, and O‐GlcNAcylation. Aberrant glycosylation has been implicated in multiple neurodegenerative conditions. This figure highlights the dysregulated expression of glycans and the abnormal glycosylation of target proteins (as discussed in this review) within these diseases. The figure was drawn via Adobe Illustrator (Adobe Inc., San Jose, CA, USA).

TDP‐43 forms cytoplasmic phosphorylated inclusions in ALS, contributing to cytotoxicity through abnormal protein‐RNA interactions [155]. O‐GlcNAc at Thr199 and Thr233 of TDP‐43 has been shown to suppress its aggregation and hyperphosphorylation, whereas loss of this modification impairs RNA splicing activity [92]. Reduced O‐GlcNAc enhances liquid–liquid phase separation (LLPS), inducing the formation of insoluble aggregates [164]. In the brains of ALS patients, TDP‐43 results in decreased O‐GlcNAcylation and increased phosphorylation, and pharmacological inhibition of OGA increases the O‐GlcNAc of TDP‐43 and reduces aggregate formation [92] (Figure 4).

FUS is a multifunctional DNA/RNA‐binding protein involved in transcription, RNA splicing, and microRNA processing [156]. FUS forms stress granule (SG)‐like aggregates in ALS [165]. Glycosylation reduces the aggregation propensity of FUS, with mucin‐type O‐glycosylation regulating its phase separation [166]. Impaired FUS glycosylation leads to abnormal SG solidification, disrupted RNA metabolism, and motor neuron death [165, 167]. Notably, certain ALS‐associated mutations (e.g., FUS‐R521C) impair glycosylation and promote pathological aggregation [168].

## 5 Clinical Potential of Glycosylations

Given the critical role of glycosylation in regulating numerous cellular signaling pathways and protein functions, inhibitors that target glycosylation hold promising therapeutic potential. However, current therapeutic development remains largely at the stage of identifying glycosylation‐related targets, and no glycosylation‐targeting inhibitors have yet received clinical approval. The clinical potential of glycosylation in neurodegenerative diseases is primarily reflected in two key areas: diagnosis and treatment [169].

In diagnostics, aberrant glycosylation patterns may serve as early biomarkers [170, 171]. The detection of specific glycosylation signatures in cerebrospinal fluid or blood—such as O‐GlcNAc levels of the tau protein or the N‐glycosylation profile of Aβ—can provide highly specific diagnostic indicators for Alzheimer's disease, Parkinson's disease, and other neurodegenerative disorders [172, 173, 174]. Advances in technologies such as mass spectrometry and lectin microarrays have enabled high‐throughput glycosylation profiling, facilitating early disease detection, precise monitoring of disease progression, and evaluation of therapeutic efficacy [175, 176].

On the therapeutic front, significant progress has been made in the development of drugs that target glycosylation pathways [177, 178]. Modulating the activity of key glycosylation enzymes—such as O‐GlcNAc transferases or glycosidases—can effectively intervene in the aberrant aggregation of pathological proteins [179, 180]. For example, OGA inhibitors help sustain the O‐GlcNAc of the tau protein, thereby inhibiting its hyperphosphorylation [181, 182]. Similarly, modulators of N‐glycosylation can influence APP processing to reduce Aβ production [183]. Several glycosylation‐targeted compounds, including Thiamet‐G, have advanced to clinical trials, demonstrating neuroprotective potential [184, 185]. Furthermore, the integration of nanocarrier drug delivery systems has significantly improved the blood–brain barrier permeability of glycosylation‐modulating agents, removing a key barrier to clinical application [186, 187]. Collectively, these advances are accelerating the translational journey of glycosylation‐targeted therapies from the bench to the bedside.

## 6 Outlook

As a critical post‐translational modification, glycosylation has emerged as a focal point in current research on neurodegenerative diseases [60]. This review systematically summarizes the mechanisms and therapeutic potential of the two most prevalent forms—N‐glycosylation and O‐glycosylation—in AD, PD, HD, and ALS.

The dynamic balance of glycosylations is essential for maintaining neuronal function [188]. Studies have demonstrated that the “yin‐yang” interplay between O‐GlcNAc and phosphorylation plays a central role in regulating the aggregation of tau and α‐synuclein proteins [189, 190]. The levels of O‐GlcNAc and its catalytic enzymes (OGT/OGA) fluctuate in response to the cellular environment and stress conditions [191, 192, 193]. Dysregulation of this dynamic balance can disrupt cellular signaling pathways, contributing to disease onset—a phenomenon that explains why the reduced O‐GlcNAc levels observed in AD and PD patients are associated with severe pathological consequences [134, 193]. However, excessive enhancement of O‐GlcNAc may also interfere with normal signaling, underscoring the importance of precisely defining the “therapeutic window”—a key challenge in future drug development [194].

Glycosylation‐targeted therapies have shown considerable clinical promise [6]. Among them, OGA inhibitors such as MK‐8719 have demonstrated significant neuroprotective effects in PD models and have entered clinical trials [148]. However, several key challenges remain: (1) Although some OGA inhibitors have advanced through preclinical stages, their safety and efficacy in clinical applications require further validation; (2) drug delivery is limited by the blood–brain barrier (BBB), necessitating the development of nanocarrier systems with improved BBB permeability; (3) the inherent complexity of glycosylation networks increases the risk of off‐target effects during therapeutic intervention; and (4) interindividual variability in glycosylation profiles may influence therapeutic responses. Future research should focus on developing more precise interventions, such as personalized treatment strategies based on patient‐specific glycosylation patterns, and on optimizing delivery systems to increase brain‐targeting efficacy.

In the future, the integration of multi‐dimensional omics technologies holds tremendous promise for deciphering the complex role of glycosylation in neurodegenerative diseases [195, 196]. By combining glycoproteomics, transcriptomics, metabolomics, and spatial omics, researchers may be able to construct detailed, cell type‐ and region‐specific glycosylation maps of the brain [197]. These high‐resolution atlases not only reveal dynamic glycosylation alterations at early disease stages but also help uncover the cellular context in which these changes occur [197, 198]. This level of insight is expected to enable precise identification of disease‐associated glycosylation events, providing a molecular foundation for early diagnosis and target discovery [199].

Another crucial direction is the in‐depth exploration of the interplay between glycosylation and other posttranslational modifications (PTMs) [50]. Glycosylation frequently co‐occurs with modifications such as phosphorylation, ubiquitination, SUMOylation, and acetylation [200]. These modifications can exert cooperative or competitive effects, forming intricate regulatory networks that influence protein function, trafficking, and degradation [201]. Dissecting the interdependency of these PTMs will provide a more comprehensive understanding of how glycosylation regulates neuronal homeostasis and contributes to disease progression. This knowledge is expected to inform the development of multitarget therapeutic strategies that modulate multiple modification pathways simultaneously.

Emerging computational technologies, especially those based on artificial intelligence and machine learning, offer new opportunities to accelerate glycosylation research [202, 203]. AI‐assisted prediction of glycosylation sites, structural modeling of glycan chains, and deep learning‐based pattern recognition can significantly increase our ability to identify clinically relevant biomarkers from large and heterogeneous datasets [204]. These approaches may also support the rational design of glycosylation‐targeted therapeutics by modeling structure–activity relationships and simulating glycoenzyme–substrate interactions. Moreover, integrating clinical data with glycomic profiles through AI may eventually enable the realization of predictive, preventive, and personalized glyco‐medicine.

Finally, the future of glycosylation‐targeted therapy lies in greater specificity, safer pharmacological profiles, and individualized application [6]. The heterogeneity of glycosylation among patients suggests that one‐size‐fits‐all approaches may be inadequate. Personalized therapeutic regimens based on glycosylation signatures could improve drug efficacy and reduce adverse effects. In parallel, early‐stage changes in glycosylation, which are detectable in cerebrospinal fluid or blood, may serve as minimally invasive biomarkers for disease surveillance [172]. At the forefront of drug development, novel OGA inhibitors, N‐glycosylation modulators, and glycan‐mimicking compounds, particularly those delivered by nanocarrier systems with enhanced blood–brain barrier penetration, represent key areas for future innovation [205]. Continued interdisciplinary collaboration will be critical to overcoming these challenges and translating glycosylation‐targeted approaches into clinical success.

## Author Contributions

Haihan Yu, Xing Chen, Mengke Gu, Yang Yang, Kaidi Ren, and Ziqing Wei conceptualized and wrote the manuscript and created the figures. Haihan Yu and Ziqing Wei contributed to the writing of the manuscript. Ziqing Wei, Haihan Yu, Yang Yang, and Kaidi Ren reviewed and modified the manuscript. All authors approved the final version of the manuscript.

## Consent

All authors have approved this manuscript for publication.

## Conflicts of Interest

The authors declare no conflicts of interest.

## Data Availability

All data generated or analyzed in this study is available from the corresponding author upon reasonable request.
